# Concordance in prediction body fat percentage of Brazilian women in reproductive age between different methods of evaluation of skinfolds thickness

**DOI:** 10.20945/2359-3997000000246

**Published:** 2020-06-05

**Authors:** Gislaine Satyko Kogure, Rafael Costa Silva, Victor Barbosa Ribeiro, Maria Célia Mendes, Rafael Menezes-Reis, Rui Alberto Ferriani, Cristiana Libardi Miranda Furtado, Rosana Maria dos Reis

**Affiliations:** 1 Faculdade de Medicina de Ribeirão Preto Departamento de Ginecologia e Obstetrícia Universidade de São Paulo Ribeirão Preto SP Brasil Faculdade de Medicina de Ribeirão Preto, Setor de Reprodução Humana, Departamento de Ginecologia e Obstetrícia, Universidade de São Paulo, Ribeirão Preto, SP, Brasil; 2 Instituto Federal de São Paulo Campus de Jacareí SP Brasil Instituto Federal de São Paulo, Campus de Jacareí, SP, Brasil; 3 Instituto de Saúde e Biotecnologia Universidade Federal do Amazonas Manaus AM Brasil Instituto de Saúde e Biotecnologia. Universidade Federal do Amazonas, Manaus, AM, Brasil; 4 Centro de Pesquisa e Desenvolvimento de Medicamentos Departamento de Cirurgia Universidade Federal do Ceará Fortaleza CE Brasil Centro de Pesquisa e Desenvolvimento de Medicamentos, Departamento de Cirurgia, Programa de Pós-Graduação em Ciências Médicas e Cirúrgicas, Universidade Federal do Ceará, Fortaleza, CE, Brasil

**Keywords:** Body fat, electric impedance, dual-energy x-ray absorptiometry, skinfold thickness

## Abstract

**Objective:**

To assess the utility of bioimpedance (BIA) and skinfolds thickness (SF) in body fat percentage measuring (%BF) compared to the reference method dual-energy x-ray absorptiometry (DXA) in Brazilian reproductive age women, as well as to estimate of inter- and intra-observer precision for SF.

**Subjects and methods:**

170 women aged 18-37 years with BMI between 18 and 39.9 kg/m^2^ were selected for this cross-sectional study. Body density was evaluated through equations proposed by Jackson, Pollock and Ward (1980) (Eq_JPW_) and Petroski (1995) (Eq_PET_), and %BF was estimated by BIA, DXA and Siri’s formula (1961). The SF were measured by two separate observers: A and B (to determine inter-observer variability), who measured the folds at three times with 10-minute interval between them (to determine intra-observer variability – we used only observer A).

**Results:**

The %BF by DXA was higher than those measured by SF and BIA (p<0.01, for all) of 90 volunteers. The Lin coefficient of agreement was considered satisfactory for %BF values obtained by Eq_JPW_ and BIA (0.55) and moderate (0.76) for sum of SF (ΣSF) values obtained by Eq_JPW_ and Eq_PET_. No agreement was observed for the values obtained by SF (Eq_JPW_ and Eq_PET_), BIA and DXA. Analysis of inter- and intra-observer of 59 volunteers showed that different measures of SF thickness met acceptability standards, as well as the % BF.

**Conclusion:**

BIA and SF measurements may underestimate %BF compared with DXA. In addition, BIA and SF measurements are not interchangeable with DXA. However, our results suggest the equation proposed by Jackson, Pollock and Ward (three skinfolds) compared to BIA are interchangeable to quantify the %BF in Brazilian women in reproductive age. Furthermore, our results show acceptable accuracy for intra- and inter-observer skinfold measurements. Arch Endocrinol Metab. 2020;64(3):257-68

## INTRODUCTION

The global obesity epidemic is a serious public health problem. Approximately, 39% of adults aged 18 years or over are overweight and 13% are obese. Women have higher rates independent of body mass index (BMI) ( 1 ). Dietary habits and physical inactivity are key drivers for excess body weight, intensified by hereditary factors. However, reproductive health factors may also contribute to adiposity in women ( 2 ). Changes in body weight may be related physiological changes due to menstrual cycle, and the use of hormonal contraceptive with water retention hypothesis and increased body fat ( 3 ). In fact, general and local fat accumulation are correlated with comorbidity and pathophysiologic processes including metabolic syndrome ( 4 ) and infertility ( 5 ). Furthermore, the body fat percentage (%BF) variability has contrasting effects on cardiovascular risk factors, while body weight variability has no significant effects on men and women ( 6 ), which highlights the importance of body composition evaluation, since BMI is insensitive to the actual distribution of body fat ( 7 ).

There are several available techniques for the assessment of body composition. DXA is a two-dimensional imaging technique that uses X-rays with two different energies, it provides a rapid and non-invasive assessment of fat mass, free-fat mass and bone mineral density, and is considered to be the reference method in clinical research ( 8 ). However, the most frequently applied models to evaluate body composition in clinical practice and epidemiology is the bicompartmental model, splits the body into fat mass (water-free body component) and fat-free mass (skeletal muscle, internal organs, and interstitial fat tissue) ( 9 ). Skinfold measurement (SF) and bioelectrical impedance analysis (BIA) are two doubly indirect common methods to examine body composition and estimate %BF and are inexpensive instrument which overcomes BMI limitations. SF thickness are based on the observation that the greatest proportion of body fat is located in the subcutaneous tissue and its can be analyzed in two ways. One of them is considering SF measurements of different anatomical regions separately and the second way is to include them in developed equations from mathematical regressions to estimate body composition in different ethnic and age groups ( 10 , 11 ). BIA measures the electrical properties of body tissues where it is possible to estimate the amount of body water and, by assuming constant values, the proportion of fat-free mass and body fat ( 9 , 12 ).

Given the above, the present study proposed to assess %BF values obtained with three different body composition techniques: SF, BIA and DXA in non-hormonal contraceptive user women, as well as to verify the agreement between the methods using DXA as gold standard. Furthermore, as the measurement of SF thickness is sensitive to inter- and intra-observer errors the article also aimed to verify inter- and intra-observer reliability.

## SUBJECTS AND METHODS

### Study population

During 2012-2013, 170 women aged 18-37 years, with BMI between 18 e 39.9 kg/m^2^, and who had not engaged in regular and systematic physical exercise were selected for this study. The volunteers were selected from the Outpatient Clinics of the Human Reproduction sector of the Department of Gynecology and Obstetrics at the University Hospital of Ribeirão Preto Medical School, University of São Paulo. The recruitment occurred on basic health clinics throughout the city and through public advertisements in the local newspaper and on regional television. The exclusion criteria included the presence of systemic diseases that altered body composition, smoking and pregnancy, as well as the use of drugs such as contraceptive hormones, anabolic steroids, thiazide diuretics and corticosteroid. Participants who did not complete the study were excluded. The protocol was approved by our Institutional Review Board at the University Hospital of the Ribeirão Preto Medical School, University of São Paulo (process number 13475/2009) and all participants provided written informed consent. The related trials for this intervention were registered in the Brazilian Clinical Trials Registry (ReBec; RBR-7p23c3).

### Anthropometry

Height and weight were recorded to the nearest 0.1 cm and 0.5 kg, respectively, using a standing anthropometer and weight scale, incorporated in a balance platform (Filizola, São Paulo, Brazil).

### Body fatness estimation

Before the evaluation, the procedures required for data collection were explained. The volunteers complied with a ten-hour fasting period prior to the evaluation. Furthermore, they having abstained from physical exercise, alcoholic beverages, coffee and caffeine-based beverages in the preceding 12 and 24 hours. Body composition measurements to determine %BF were made using each of these three following methods:

### Dual-energy x-ray absorptiometry (DXA)

DXA scanning (Hologic 4500 device QDR Discovery^®^ Series – Waltham: MA, USA) was also used for body composition analysis with full body scan. The analysis was performed using the 5 Discovery Wi model software (S/N 84826) version 13.0:5 (Waltham: MA, USA). The examination was conducted by experts in the Image Science Center and Medical Physics of the University Hospital, Ribeirão Preto Medical School, following the recommendations of ISCD (International Society for Clinical Densitometry) ( 13 ). The region of interest (ROI) from the scan used in this analysis was percentage fat (fat mass/total mass × 100).

### Bioelectrical impedance analysis (BIA)

To determine the estimated %BF, the tetrapolar bioimpedance test was performed by the apparatus Biodynamics, model 310e. For this, the volunteers were positioned in supine on an isolated electric conductors’ stretcher and kept in position for 5 minutes, without using any metallic object or adornment (earrings, bracelets, rings, piercings, etc). The BIA analyzer unit had 4 electrodes. Two electrodes were placed on the right hand with one just proximal to the third metacarpo-phalangeal joint, and the other near to the ulnar head. Two other electrodes were placed on the dorsal surface on the right foot with one just proximal to the third metatarsophalangea joint (positive) and the other one between the medial and lateral malleoli ( 14 ). The BIA test were performed in the Laboratory of Gynecology and Obstetrics of the Ribeirão Preto Medical School, University of São Paulo.

### Skinfold assessment

The skinfolds were measured at the Centre of Physical Education, Recreation, and Sports at the University of São Paulo. The measurements were performed on the same day in the morning period. Body measurements were taken on the right side of the body. Skinfold (SF) thickness measurements were performed at standard sites (triceps (TSF), subscapular (SSF), suprailiac (SISF), average axillary (AASF), medial thigh (MTSF) and medial calf (CSF)) using Sanny®skinfold calipers (Sanny, Brazil). The three measurements average of each SF was used in the analysis. The SF measures were used in two equations of age and gender to determine body density: equation proposed by Jackson, Pollock e Ward (Eq_JPW_) [1.0994921 - 0.0009929 (TSF + SISF + MTSF) + 0.0000023 (TSF + SISF + MTSF)^2^ - 0.0001392 (age)] ( 10 ) and equation proposed by Petroski (Eq_PET_) [D = 1.19547130 - (0.07513507 * Log10 (AASF + SISF + MTSF + CSF)) - ( 0.00041072 (Age)) ( 11 ). The choice of predictive equations took into account the gender, age and level of training. The first ( 10 ), was designed to evaluate Caucasian non-athlete women aged 18-55 years, and the second ( 11 ) counted in their sample, with a Brazilian population of non-athletes, covering a wide age group between 18 and 66 years. The absolute values or the sum of skinfold thickness (∑SF) of each protocol were calculated as a subcutaneous adipose tissue indicator. Body density was used to estimate %BF as per the Siri ( 15 ) equation: [%BF = (495 / body density) – 450].

The skinfolds were measured by two separate observers A and B (to determine inter-observer variability). They measured the folds at three times with a 10-minute interval between them (to determine intra-observer variability), in sequential order: First – Measurement of SF by observer A: A1; Second – Measurement of SF by observer B; Third – Measurement of SF by observer A: A2.

For the analysis coefficients of agreement intra-methods was used the measurements of observer A. For the analysis of the inter- and intra-observer variability, we used the Eq_JPW_ of prediction of body density by skinfolds. The calibration of the skinfold compass was performed to begin the pilot observers training with 15 patients on one-month period before starting the data collection

### Statistical analysis

Sample size was estimated to allow reasonable accuracy – defined as the 95% confidence interval width ≤ 0.20 ( 16 ). The observed value for the intraclass correlation coefficient (ICC) must be considered above the minimum required value (ICC > 0.70) for the method to be suitable for research or clinical practice ( 17 ). Considering ICC ≥ 0.70, it would be necessary to evaluate 100 subjects to have a 95% CI width ≤ 0.20. Descriptive statistics included mean values, standard deviations, median and maximum and minimum values for all analyzed variables. Statistical analyses were performed using SAS^®^ 9.0 (SAS Institute Inc., University of North Carolina, Cary, NC). To check the agreement of the methods with DXA was proposed coefficient St Laurent ( 18 ). The coefficient of St. Laurent can vary between -1 and 1, and the result closer to 1 indicates an excellent agreement between methods. To verify reproducibility (measurements between A1 and B, inter-observers) and repeatability (measurements between A1 and A2, intra-observer), was used the Concordance Correlation Coefficient (CCC) proposed by Lin ( 19 ). The CCC measures the coincidence of the regression line of the data with the perfect concordance line (45 degrees) and combines a precision component (the Pearson correlation coefficient) and one of accuracy. When the Lin coefficient is equal to one, it means that the regression line lies exactly on the line of perfect agreement. Bland and Altman graphs were also used to complement the agreement analysis of the different methods and the different observers ( 20 ). Bland and Altman strategy includes building a correlation graph (difference vs. average) and calculating the correlation threshold. With this technique it is possible to visually evaluate the agreement and the magnitude of the differences with the 95.0% confidence interval for the observations.

## RESULTS

In the present study, 170 volunteers were recruited. Fifty volunteers in the study did not meet the inclusion criteria, and 30 did not complete all body fat evaluations. Accordingly, the data from 90 volunteers on reproductive period were included in the analysis with complete data for all body fat estimation methods. Descriptive statistics for characteristics and of methods for estimate body fat of the volunteers are presented in Table 1 . For the inter- and intra-observer analyzes about body fat estimated by SF thickness variable, 31 participants in the study did not complete all evaluations on the same day. Therefore, 59 volunteers were investigated. Descriptive statistics for characteristics and the inter- and intra-observer analyze for body fat estimated by SF thickness are presented in Table 2 . The %BF by DXA was higher than those measured by SF thickness and BIA (Mean (SD)) (SF-Eq_PET_ vs DXA -5.5(6.8), SF-Eq_JPW_ vs DXA -14.9(9.2), BIA vs DXA -6.1(4.7); p < 0.01 for all).

**Table 1:** Descriptive values. Inter-method evaluation sample (n = 90)

	Mean	SD	Minimum	Median	Maximum
Age (years)	29.06	4.96	18.33	30.04	37.77
Weight (kg)	69.92	15.98	42.20	67.00	115.00
Height (m)	1.61	0.05	1.46	1.61	1.75
BMI (kg/m^2^)	26.86	5.92	16.08	26.02	41.43
Average axillary SF	17.64	7.92	5.00	17.00	41.50
Tricipital SF	20.13	6.81	8.00	18.05	40.10
Suprailiac SF	25.07	10.04	9.50	24.75	48.00
Medial thigh SF	35.08	10.39	15.00	35.00	63.10
Calf SF	22.28	7.42	7.20	21.90	39.00
∑ SF – Eq_JPW_	90.16	34.06	34.50	85.50	203.50
∑ SF – Eq_PET_	100.06	30.70	49.80	97.70	171.50
Body density – Eq_JPW_	1.03	0.02	0.99	1.03	1.06
Body density – Eq_PET_	1.05	0.01	1.03	1.05	1.07
% BF – Eq_JPW_	32.00	8.07	15.44	31.97	50.76
% BF – Eq_PET_	22.52	4.68	13.08	22.96	31.27
% BF – DXA	37.43	6.46	19.00	38.15	52.60
% BF – BIA	32.01	7.08	12.30	33.10	44.00

SD: standard deviation; BMI: body mass index; SF: skinfolds; ∑SF: sum of skinfolds; %BF: body fat percentage; DXA: dual-energy x-ray absorptiometry; BIA: bioelectrical impedance analysis.

**Table 2:** Descriptive values. Inter- and intra-observer’s evaluation sample (n = 59)

	Mean	SD	Minimum	Median	Maximum
Age (years)	29.28	4.79	19.78	30.28	37.75
Weight (kg)	68.00	13.30	42.20	65.00	102.20
Height (m)	1.62	0.05	1.51	1.62	1.75
BMI (kg/m^2^)	25.91	4.90	16.08	25.66	36.84
Tricipital SF – Observer A1	19.45	6.46	8.00	17.00	36.10
Tricipital SF – Observer B	19.00	6.55	7.20	17.00	35.00
Tricipital SF – Observer A2	19.55	6.43	8.50	17.30	36.30
Suprailiac SF – Observer A1	25.59	11.20	9.50	23.50	48.00
Suprailiac SF – Observer B	29.27	12.30	8.50	28.50	53.50
Suprailiac SF – Observer A2	25.72	11.23	9.50	23.80	48.20
Medial thigh SF – Observer A1	34.37	9.89	16.00	35.00	57.00
Medial thigh SF – Observer B	34.56	11.36	15.00	33.00	57.20
Medial thigh SF – Observer A2	34.55	9.94	16.10	35.30	57.30
∑ SF – Observer A1	79.41	24.99	34.50	79.10	135.00
∑ SF – Observer B	82.82	27.89	31.70	82.00	139.50
∑ SF – Observer A2	79.82	25.01	35.30	79.60	135.60
% BF – Observer A1	29.53	7.23	15.01	30.12	43.64
% BF – Observer B	30.38	7.87	13.98	30.81	44.44
% BF – Observer A2	29.65	7.22	15.30	30.26	43.75
Body density – Observer A1	1.03	0.02	1.00	1.03	1.06
Body density – Observer B	1.03	0.02	1.00	1.03	1.07
Body density – Observer A2	1.03	0.02	1.00	1.03	1.06

SD: standard deviation; BMI: body mass index; SF: skinfolds; ∑SF: sum of skinfolds; %BF: body fat percentage; DXA: dual-energy x-ray absorptiometry; BIA: bioelectrical impedance analysis.

**Figure 1:**
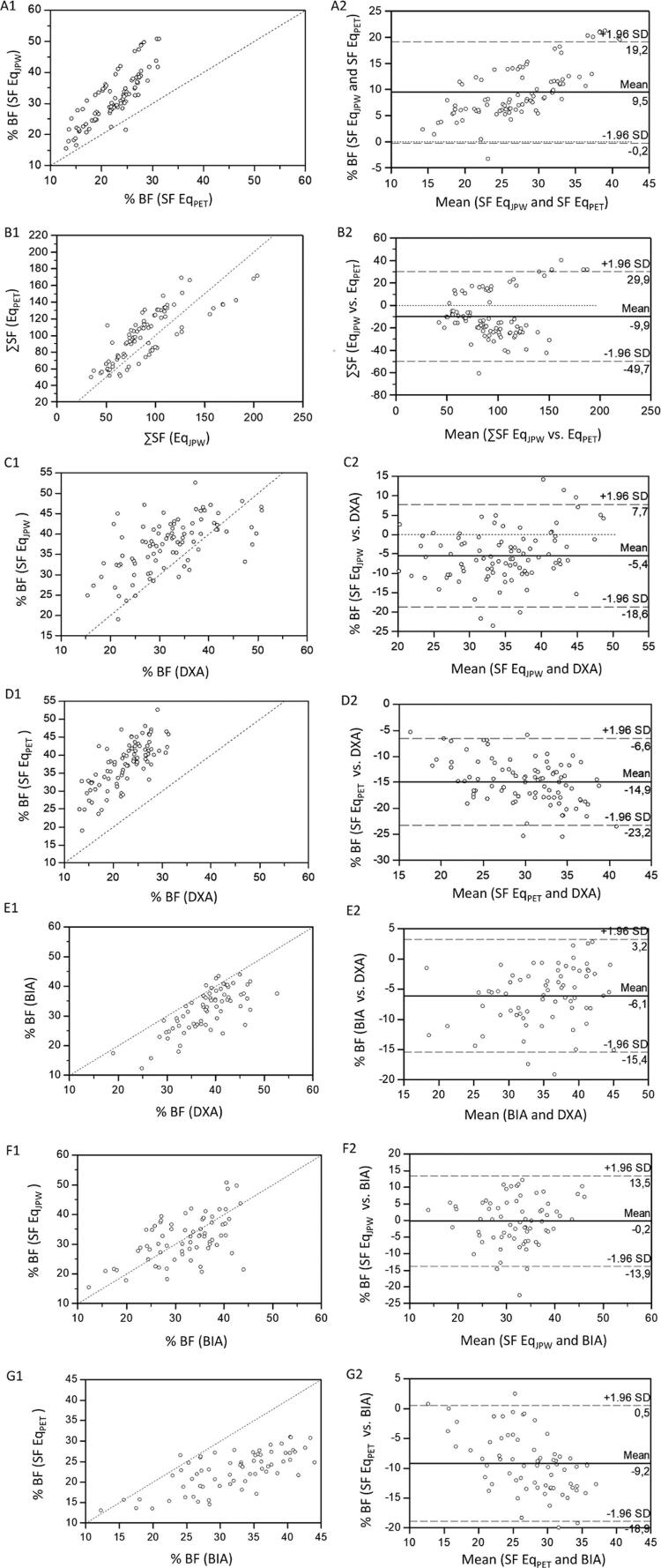
Scatter plots showing agreement between the methods used to estimate %BF – (A1) SF EqJPW vs. SF EqPET, (B1) ∑SF EqJPW vs. ∑SF EqPET, (C1) SF EqJPW vs. DXA, (D1) SF EqPET vs. DXA, (E1) BIA vs. DXA, (F1) SF EqJPW vs. BIA, (G1) SF EqPET vs. BIA. The straight line shows the expected linear relationship and the scattered points around the line show how the actual data diverge from the expected, and Bland-Altman plots showing the limits of agreement between the methods used to estimate %BF – (A2) SF EqJPW vs. SF EqPET, (B2) ∑SF EqJPW vs. ∑SF EqPET, (C2) SF EqJPW vs. DXA, (D2) SF EqPET vs. DXA, (E2) BIA vs. DXA, (F2) SF EqJPW vs. BIA, (G2) SF EqPET vs. BIA. The center line represents the mean differences between the two observers, and the other two lines represent two SDs from the mean. SDs: standard deviation(s); BIA: bioelectrical impedance analysis; DXA: dual energy X-ray absorptiometry; SF: skinfold thickness; Eq_JPW_: equation proposed by Jackson, Pollock e Ward (1980); Eq_PET_: equation proposed by Petroski (1995); ∑: sum; vs: versus.

The agreement coefficient values between SF thickness and BIA analysis to DXA are presented in Table 3 . No agreement was observed between DXA and the other methods (BIA and SF thickness). However, we observed that Lin’s coefficient of agreement was considered satisfactory for %BF values obtained by Eq_JPW_ and BIA (0.55) and moderate (0.76) for sum of SF (ΣSF) values obtained by Eq_JPW_ and Eq_PET_. Figure 1 (A1-G1) shows the scatter plots of the methods used to estimate %BF and Figure 1 (A2-G2) shows agreement limits of the relative difference between anthropometric equations and DXA-measured and BIA by Bland-Altman plots.

**Table 3:** Intermethods and Inter- and intra-observer variance

	CCC	95 (CI)	

		LI	LS
**Intermethods variance. Lin’s concordance correlation coefficient (LCC)**			
% BF – Eq_JPW_ vs. Eq_PET_	0.352	0.269	0.430
∑ SF – Eq_JPW_ vs. Eq_PET_	0.768	0.674	0.837
% BF – Eq_JPW_ vs. BIA	0.553	0.367	0.697
% BF – Eq_PET_ vs. BIA	0.299	0.200	0.391
**Intermethods variance. Coefficient of agreement for St. Laurent**			
% BF – Eq_JPW_ vs. DXA	0.360	0.262	0.440
% BF – Eq_PET_ vs. DXA	0.148	0.106	0.182
% BF – BIA vs. DXA	0.371	0.260	0.460
**LCCC. Inter-observer (first observer versus second observer measurements)**			
Tricipital SF	0.988	0.981	0.993
Medial thigh SF	0.898	0.843	0.935
Suprailiac SF	0.900	0.842	0.938
∑ SF	0.952	0.924	0.970
% BF	0.960	0.936	0.975
**LCCC. Intra-observer variance (single observer first versus second measurements)**			
Tricipital SF	0.999	0.999	0.999
Medial thigh SF	0.999	0.999	0.999
Suprailiac SF	0.999	0.999	0.999
∑ SF	0.999	0.999	0.999
% BF	0.999	0.999	0.999
Tricipital SF	0.999	0.999	0.999

CCC: concordance correlation coefficient; LCCC: Lin’s concordance correlation coefficient; SF: skinfolds; CI: confidence interval; SD: standard deviation; IL: inferior limit; UL: upper limit; ∑SF: sum of skinfolds; % BF: body fat percentage; DXA: dual-energy x-ray absorptiometry; BIA: bioelectrical impedance analysis.

**Figure 2:**
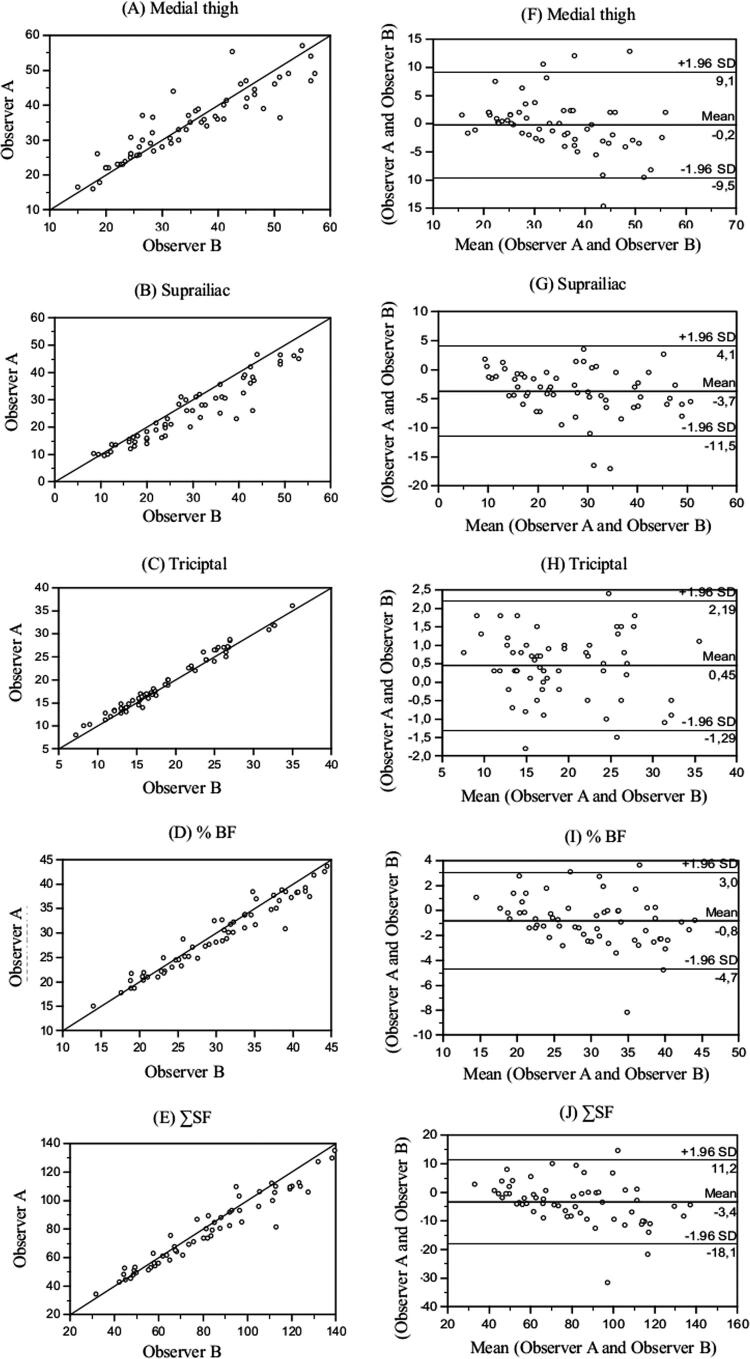
Interobserver. Scatter plots (A-E) showing agreement between medial thigh, suprailiac, triciptal, %BF and ∑SF as determined by skinfold using. The straight line shows the expected linear relationship and the scattered points around the line show how the actual data diverge from the expected, and Bland-Altman (F-J) plots showing the limits of agreement between medial thigh, suprailiac, triciptal, %BF and ∑SF as determined by skinfold using the measurements of observer A and observer B. The center line represents the mean differences between the two observers, and the other two lines represent two SDs from the mean. SDs: standard deviation(s); %BF: body fat percentage; SF: skinfold thickness; ∑: sum; vs: versus.

**Figure 3:**
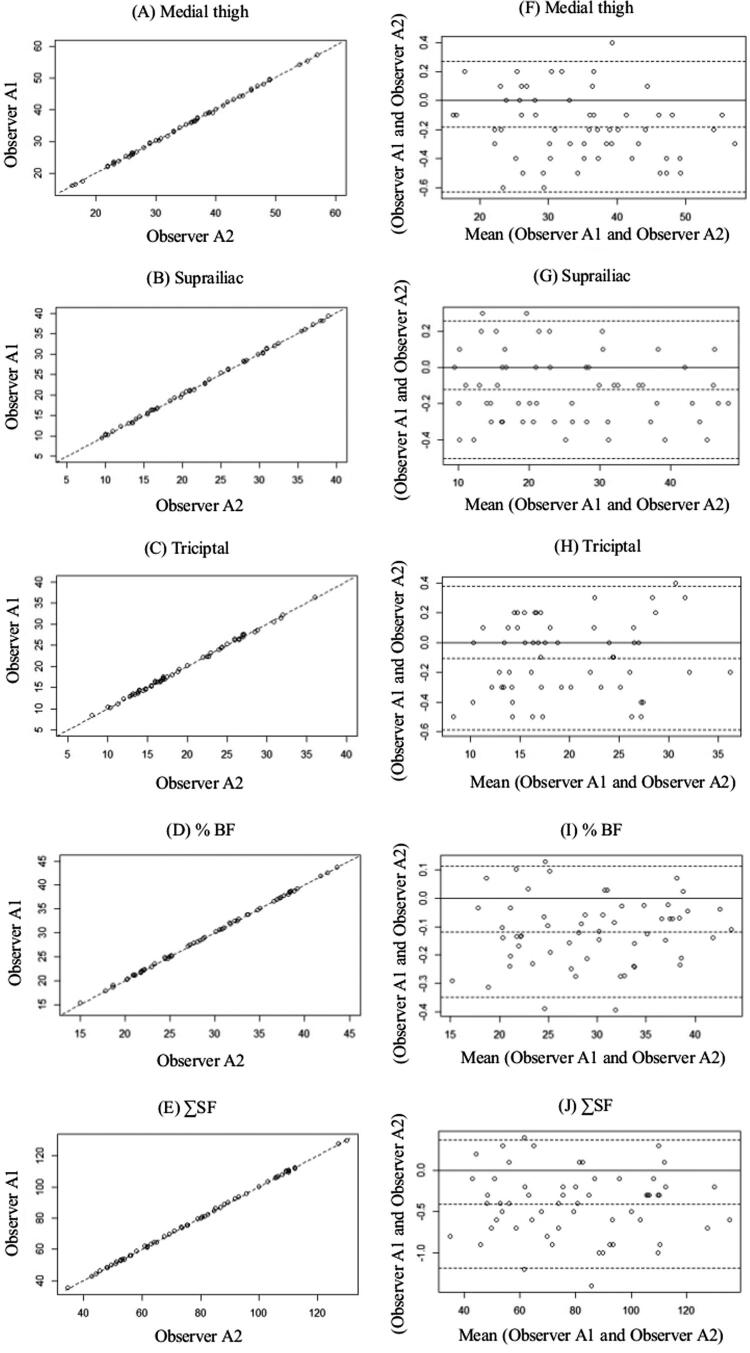
Intra-observer. Scatter plots (A-E) showing agreement between medial thigh, suprailiac, triciptal, %BF and ∑SF as determined by skinfold using. The straight line shows the expected linear relationship and the scattered points around the line show how the actual data diverge from the expected, and Bland-Altman (F-J) plots showing the limits of agreement between medial thigh, suprailiac, triciptal, %BF and ∑SF as determined by skinfold using the measurements of observer A and observer B. The center line represents the mean differences between the two observers, and the other two lines represent two SDs from the mean. SDs: standard deviation(s); %BF: body fat percentage; SF: skinfold thickness; ∑: sum; vs: versus.

The inter-observer analysis showed that the different measures of SF met acceptability standards ( Table 3 ). TSF showed greater concordance among the observers, followed by MTSF and SISF, respectively. The ∑SF and % BF also showed an excellent concordance. Figure 2 (A-E) shows agreement between inter-observers by Scatter plots, and Figure 2 (F-J) shows agreement limits of the relative difference inter-observer by Bland-Altman plots. Interobserver analysis showed that the different measures of SF thickness met acceptability standards, as well as the ∑SF and %BF ( Table 3 ). Figure 3 (A-E) shows agreement between intra-observer by Scatter plots, and Figure 3 (F-J) shows agreement limits of the relative difference intra-observer by Bland-Altman plots.

## DISCUSSION

In this study, we assessed the agreement of %BF estimates obtained from anthropometrics equations and BIA using DXA-measured %BF as the criterion in reproductive age and not users of hormonal contraceptive women. As well as, we observed whether there are differences in SF measurements in intra- and inter-observer reliability. The main findings of the study were the weakness in %BF estimating of SF thickness anthropometric predictive equations proposed by Petroski and Jackson, Pollock and Ward, and BIA as when compared to DXA. As well as, the equation proposed by Jackson, Pollock and Ward showed moderated concordance correlation with BIA, and very good reliability for inter- and intra-observer SF measurements, respectively.

We observed differences between the studied methods (DXA, BIA and SF) to assess body fat. These differences were previously reported with different equations to estimate %BF. In both male and female college age students ( 21 ), the BIA and SF thickness measurements underestimated DXA values, with a greater discrepancy for SF. In women with pre- and post-menopausal status ( 22 ), the BIA and SF values considerably underestimated the DXA values, although it was observed a significant correlation between the methods. In overweight and obese Brazilian women Braulio and cols. ( 23 ) observed that only one of the three equations studied for BIA underestimated the %BF compared to DXA, with no differences for SF thickness. Furthermore, different from our results, using a specific equation for obesity, BIA was the method that better agreed with DXA with a Lin coefficient of agreement of 0.9407. In a large cut (male and female) matched for BMI, a study showed that BIA underestimated FM compared to the DXA method. Moreover, observed the lack of agreement between the BIA and DXA methods independently of the BMI ( 12 ). A recent study compared various methods of tracking body composition across a college women’s basketball season and showed that BIA provided comparable agreement and, with SF methods having lower agreement including equation proposed by Jackson, Pollock and Ward ( 24 ).

The agreement between BIA and DXA varies, and the disagreement degree also varies substantially based on body size and sex. Tinsley and cols. ( 25 ) observed that bias magnitude was greater in women and on subjects with smaller amount of fat mass, indicating that the BIA underestimates fat mass in relation to DXA; on the other hand, in individuals with higher fat mass, the concordance was better. Recent studies showed that in both male and female with BMI < 16, BIA overestimated fat mass by 2.57 kg and for BMI > 18.5 and BMI < 40, BIA underestimated fat mass from 2.51 to 5.67 kg compared with DXA method ( 12 ). In additional, DXA uses a 3-compartment model (bone, protein/muscle, and fat) compared to BIA and SF that uses a 2-compartment model (fat-free mass, fat mass). In addition, DXA was first developed to evaluate bone mass ( 26 ). However, it is also show excellent agreement with magnetic resonance imaging (MRI) for measure whole-body adipose tissue or fat and lean tissue ( 8 ).

To our knowledge, there have been no previous comparison and agreement of %BF in reproductive age women in through SF by Eq_JPW_ and Eq_PET_ and DXA. Previous study, investigated the %BF non-pregnant women with mean age of 27.58 ± 13.76 years and concluded that BMI, anthropometric indices and SF methods of Eq_PET_ and EqJ_PW_ (but not BIA) were the most effective to assess a body fat ( 27 ). In our study, both Eq_JPW_ and Eq_PET_ underestimated the %BF by DXA, with worse values for Eq_PET_. In addition, our results of agreement were considered satisfactory for %BF values obtained by Eq_JPW_ and BIA (0.55). The Eq_JPW_ based on 3 SF which represents subcutaneous adipose tissue distribution in the whole body: upper limbs (triceps); trunk (suprailiac) and lower limb (medial thigh) to compared to Eq_PET_ based only on trunk (average axillary and suprailiac) and lower limb (medial thigh and medial calf), suggesting that the choice of the SF for the composition of the equations can be one of the factors involved in the origin of the discrepancies between the results ( 28 , 29 ). Ball and cols. ( 30 ) investigated the accuracy of the Eq_JPW_ for predicting %BF in women, using DXA as the criterion measure, and observed underestimation of the results by SF equation. In this study, the sample was similar to Jackson and cols. (1980) where the Eq_JPW_ 3 skinfold were developed. According to Guedes and Guedes (2003) ( 31 ) the equations proposed by Jackson, Pollock and Ward (1980) has a smaller prediction error within the tolerable limits and variety of age groups.

In addition, the regression equations of SF and BIA, the different equipment for each methodology and the different populations explain the diversity of results on various studies. It is decisive to choose a specific population formula suitable to convert body density to body fat percentage. The indiscriminate use of equations based on SF thickness, without validity or based on different populations, cause immeasurable errors of estimation of body composition ( 11 ). The BIA equations developed in a specific population are only generalizable for similar populations and caution is required when applying to a population other than the validation sample in order to avoid inaccurate results and erroneous interpretations ( 12 ). However, when the appropriate test protocols are followed, the BIA test accuracy is similar to the skinfold test ( 32 ).

Acceptable intra-observer agreement was achieved for all the skinfold thickness measurements. In inter-observer, the triceps skinfold presented the highest agreement. Arroyo et al (2010) ( 33 ) observed intra-observer variability acceptable between twenty-six dietitians in 10 volunteers (> 20 years) of both genders in skinfold thicknesses (triceps and biceps). In addition, the authors observed a higher variation on the relative technical error of measurement for biceps SF than for triceps SF. Although we do not evaluate the standard error of measurements, a possible cause for our results for inter-observer would be the higher values were relative measurement technical error happen in regions of higher fat accumulation ( 34 ), that is higher for medial thigh and suprailiac SF than for triceps SF. In additional, inter-observer errors seem to be the most problematic, with inadequate choice of SF site, causing the greatest variation among observers ( 35 ), and obesity may influence the skinfold measurements reliability, especially in those cases in which skinfold size approaches the upper limit of the measurement range of the caliper ( 36 ). In other studies, compared with female subjects, the inter- and intra-observer variabilities were both greater on the male subjects for %BF was obtained using the ∑SF sites (bicep, triceps, subscapular, and suprailiac) ( 37 ). The lower the variability between repeated measurements on the same subject by one (intra-observer differences), two or more (inter-observer differences) observers, the higher the precision ( 36 ).

There are limitations in the current study that need to be mentioned. First, the sample size was not sufficient to verify the agreement in BF% between the methods distinct SF and BIA using DXA as gold standard. In addition, the small sample size rendering a difficult on the subgroup’s analysis (normal BMI, overweight and obese). Furthermore, we did not have standardized the menstrual cycle phase for the %BF evaluation. However, previous studies have observed that hormonal fluctuations occurring during the menstrual cycle do not alter the body composition measured by BIA ( 38 ), and also were not associated with subcutaneous adipose tissue change ( 39 ). Another limitation is an impossibility to fully control the correct implementation of the BIA protocol, because there are procedures and restrictions that arrive 48 hours before the test, which makes it impossible to follow the volunteers.

The main strengths of our study are that all anthropometric measurements have been performed under the same conditions by the same observers, in order to minimize a possible technical error. Furthermore, our data have been compared to DXA. The DXA measurements are greatly reproducible and their validity has been previously demonstrated ( 8 ), and reliability levels are well accepted for the development and validation on doubly indirect methods, such as anthropometry ( 40 )_._ To the best of our knowledge, this study is the first to examine the agreement between SF thickness equations and BIA to assess %BF compared to DXA, in reproductive age and nonusers of hormonal contraceptive women, as well as the reliability and reproducibility of skinfold inter- and intra-observer.

In conclusion, BIA and SF measurements may underestimate %BF compared with DXA. Our study also reported the lack of concordance between BIA and DXA methods, as well as SF (Eq_JPW_ and Eq_PET_) and DXA method. However, the equation proposed by Jackson, Pollock and Ward (three skinfolds) compared to BIA were interchangeable to quantify the %BF, but not the equation proposed by Petroski in Brazilian women in reproductive age. In addition, our results show acceptable accuracy for intra- and inter-observer skinfold measurements. Therefore, we recommend trained and experienced evaluators with the objective of controlling and minimizing anthropometric measurement error and the results obtained when calculating %BF from skinfold measurements. Future investigations are needed to evaluate the use of these methods in a larger cohort of Brazilian women of reproductive age in BMI categories.

## References

[1] WHO. Obesity and overweight [Internet]. Fact sheet N 311. 2018 [cited 2019 Nov 24]. Available from: http://www.who.int/news-room/fact-sheets/detail/obesity-and-overweight.

[2] Trikudanathan S, Pedley A, Massaro JM, Hoffmann U, Seely EW, Murabito JM, et al. Association of female reproductive factors with body composition: the Framingham Heart Study. J Clin Endocrinol Metab [Internet]. 2013;98(1):236-44. 10.1210/jc.2012-1785 PMC3537091 23093491

[3] Clark MK, Dillon JS, Sowers M, Nichols S. Weight, fat mass, and central distribution of fat increase when women use depot-medroxyprogesterone acetate for contraception. Int J Obes. 2005;29(10):1252-8. 10.1038/sj.ijo.0803023 15997247

[4] Baudrand R, Domínguez JM, Tabilo C, Figueroa D, Jimenez M, Eugenin C, et al. The estimation of visceral adipose tissue with a body composition monitor predicts the metabolic syndrome. J Hum Nutr Diet. 2013;26(Suppl. 1):154-8. 10.1111/jhn.12089 23634931

[5] Sugiyama T, Watanabe H, Takimoto H, Fukuoka H, Yoshiike N, Sagawa N. Management of Obesity in Pregnancy. Curr Womens Health Rev [Internet]. 2009;5(4):220-4.

[6] Saito Y, Takahashi O, Arioka H, Kobayashi D. Associations between body fat variability and later onset of cardiovascular disease risk factors. PLoS One. 2017;12(4):e0175057. 10.1371/journal.pone.0175057 PMC5378370 28369119

[7] Thomas EL, Frost G, Taylor-Robinson SD, Bell JD. Excess body fat in obese and normal-weight subjects. Nutr Res Rev. 2012;25: 150-61. 10.1017/S0954422412000054 22625426

[8] Borga M, West J, Bell JD, Harvey NC, Romu T, Heymsfield SB, et al. Advanced body composition assessment: from body mass index to body composition profiling. J Investig Med. 2018 Jun;66(5):1-9. 10.1136/jim-2018-000722 PMC5992366 29581385

[9] Marra M, Sammarco R, De Lorenzo A, Iellamo F, Siervo M, Pietrobelli A, et al. Assessment of body composition in health and disease using bioelectrical impedance analysis (BIA) and dual energy x-ray absorptiometry (DXA): A critical overview. Contrast Media Mol Imaging. 2019;2019:3548284. 10.1155/2019/3548284 PMC6560329 31275083

[10] Jackson AS, Pollock ML, Ward A. Generalized equations for predicting body density of women. Med Sci Sports Exerc. 1980;12(3):175-81. 7402053

[11] Petroski EL. Development and validation of equations to estimate body density in adults. Santa Maria: UFSM. 1995. p. 124.

[12] Achamrah N, Colange G, Delay J, Rimbert A, Folope V, Petit A, et al. Comparison of body composition assessment by DXA and BIA according to the body mass index: A retrospective study on 3655 measures. PLoS One. 2018;13(7):e0200465. 10.1371/journal.pone.0200465 PMC6042744 30001381

[13] Lewiecki EM, Gordon CM, Baim S, Leonard MB, Bishop NJ, Bianchi ML, et al. International Society for Clinical Densitometry 2007 Adult and Pediatric Official Positions. Bone. 2008;43(6): 1115-21. 10.1016/j.bone.2008.08.106 18793764

[14] Lukaski HC, Bolonchuk WW, Hall CB, Siders WA. Validation of tetrapolar bioelectrical Impedance method to assess body composition. J Appl Physiol. 1986;60:1327-32. 10.1152/jappl.1986.60.4.1327 3700310

[15] Siri WE. Body composition from fluid spaces and density: analysis of methods. In: Brozek J, Henschel A, editors. Techniques for measuring body composition. Washington: National Academy of Sciences – National Research Council. 1961. p. 223-44.

[16] Bonett DG. Sample size requirements for estimating intraclass correlations with desired precision. Stat Med. 2002;21(9):1331-5. 10.1002/sim.1108 12111881

[17] Kottner J, Audige L, Brorson S, Donner A, Gajewski BJ, Hroóbjartsson A, et al. Guidelines for Reporting Reliability and Agreement Studies (GRRAS) were proposed. Int J Nurs Stud. 2011;48(6):661-71. 10.1016/j.ijnurstu.2011.01.016 21514934

[18] St Laurent RT. Evaluating agreement with a gold standard in method comparison studies. Biometrics. 1998;54(2):537-45. 9629642

[19] Lin LI-K. A Concordance Correlation Coefficient to Evaluate Reproducibility. Biometrics. 1989;45(1):255. 2720055

[20] Bland JM, Altman DG. Statistical methods for assessing agreement between two methods of clinical measurement. Lancet (London, England). 1986;1(8476):307-10. 2868172

[21] Grove J, Hung Y. Body fat prediction equations for skinfold and bioelectrical impedance analysis using dual-energy x-ray absorptiometry data as the criterion. J Phys Ther Sport Med. 2017;1(1):5-11.

[22] Sillanpää E, Häkkinen A, Häkkinen K. Body composition changes by DXA, BIA and skinfolds during exercise training in women. Eur J Appl Physiol. 2013;113(9):2331-41. 10.1007/s00421-013-2669-9 23748419

[23] Braulio VB, Furtado VCS, Silveira MDG, Fonseca MH, Oliveira JE. Comparison of body composition methods in overweight and obese Brazilian women. Arq Bras Endocrinol Metabol. 2010;54(4):398-405. 10.1590/s0004-27302010000400009 20625652

[24] Ploudre A, Arabas JL, Jorn L, Mayhew JL. Comparison of techniques for tracking body composition changes across a season in college women basketball players. Int J Exerc Sci. 2018;11(4):425-38. 10.70252/OXVM9894 PMC5955327 29795734

[25] Tinsley GM. Proportional bias between dual-energy x-ray absorptiometry and bioelectrical impedance analysis varies based on sex in active adults consuming high- and low-carbohydrate diets. Nutr Res. 2017;42:85-100. 10.1016/j.nutres.2017.05.003 28633874

[26] Doña E, Olveira C, Palenque FJ, Porras N, Dorado A, Martín-Valero R, et al. Body composition measurement in bronchiectasis: Comparison between bioelectrical impedance analysis, skinfold thickness measurement, and dual-energy x-ray absorptiometry before and after pulmonary rehabilitation. J Acad Nutr Diet. 2018;118(8):1464-73. 10.1016/j.jand.2018.01.013 29656933

[27] De Oliveira MH, Silva JCF, Ferreira RC. Comparison of Methods for Assessing Body Composition in Women. FASEB J. 2017;31(1 Supplement):151.6-151.6.

[28] Neves EB, Ripka WL, Ulbricht L, Stadnik AMW. Comparação do percentual de gordura obtido por bioimpedância, ultrassom e dobras cutâneas em adultos. Rev Bras Med do Esporte. 2013;19(5):323-7.

[29] Krueger E, Ulbricht L, Ripka W, Neves EB. Avaliação da tecnologia do ultrassom portátil e sua correlação com o percentual de gordura obtido pelas dobras cutâneas em adultos jovens. Rev Bras Ciências da Saúde – USCS. 2015;13(46):78-83.

[30] Ball SD, Altena TS, Swan PD. Comparison of anthropometry to DXA: A new prediction equation for men. Eur J Clin Nutr. 2004;58(11):1525-31. 10.1038/sj.ejcn.1602003 15162135

[31] Guedes DP, Guedes JERP. Controle do Peso Corporal. Composição Corporal. Atividade Física e Nutrição. 2. ed. Rio de Janeiro: Shape; 2003.

[32] Dehghan M, Merchant AT. Is bioelectrical impedance accurate for use in large epidemiological studies? Nutrition Journal. 2008;7:26. 10.1186/1475-2891-7-26 PMC2543039 18778488

[33] Arroyo M, Freire M, Ansotegui L, Rocandio AM. Intraobserver error associated with anthropometric measurements made by dietitians. Nutr Hosp. 2010;25(6):1053-6. 21519782

[34] Marks GC, Habicht J-P, Mueller WH. Reliability, Dependability, and Precision of Anthropometric Measurements: the Second National Health and Nutrition Examination Survey 1976-1980. Am J Epidemiol. 1989;130(3):578-87. 10.1093/oxfordjournals.aje.a115372 2764002

[35] Pollock ML, Jackson AS. Research progress in validation of clinical methods of assessing body composition. Med Sci Sports Exerc. 1984;16(6):606-15. 6392815

[36] Stomfai S, Ahrens W, Bammann K, Kovács, Mårild S, Michels N, et al. Intra- And inter-observer reliability in anthropometric measurements in children. Int J Obes. 2011;35:S45-51. 10.1038/ijo.2011.34 21483422

[37] McRae MP. Male and female differences in variability with estimating body fat composition using skinfold calipers. J Chiropr Med. 2010;9(4):157-61. 10.1016/j.jcm.2010.07.002 PMC3206567 22027106

[38] Cumberledge EA, Myers C, Venditti JJ, Dixon CB, Andreacci JL. The Effect of the Menstrual Cycle on Body Composition Determined by Contact-Electrode Bioelectrical Impedance Analyzers. Int J Exerc Sci. 2018;11(4):625-32. 10.70252/GVOS2163 PMC5841670 29541335

[39] Huaina J, Andrade C, Monteiro A, Thalia F, Amorim R, Lustosa RP, et al. Analysis of subcutaneous adiposity during the menstrual cicle. Rev do Dep Educ Física e Saúde e do Mestr em Promoção da Saúde da Univ St Cruz do Sul/Unisc. 2017;18(2):83-7.

[40] Rech CR, dos Santos DL, da Silva JCN. Development and validation of anthropometric equations for prediction of the body fat in women aged 50 to 75 year. Rev Bras Cineantropom Desempenho Hum. 2006;8(1):5-13.

